# SP1 and RARα regulate *AGAP2* expression in cancer

**DOI:** 10.1038/s41598-018-36888-x

**Published:** 2019-01-23

**Authors:** Yegor Doush, Arif A. Surani, Amaia Navarro-Corcuera, Stephanie McArdle, E. Ellen Billett, Cristina Montiel-Duarte

**Affiliations:** 1 College of Science and Technology, Nottingham Trent University, Nottingham, UK; 2 Department of Biochemistry and Genetics, University of Navarra, 31008 Pamplona, Spain; 3 The John van Geest Cancer Research Centre, Nottingham Trent University, Nottingham, UK

## Abstract

AGAP2 (Arf GAP with GTP-binding protein-like domain, Ankyrin repeat and PH domain 2) isoform 2 is considered a proto-oncogene, but not much is known about *AGAP2* gene expression regulation. To get some insight into this process, *AGAP2* proximal promoter was cloned and characterised using reporter assays. We have identified SP1 as a transcription factor bound to *AGAP2* promoter and required for *AGAP2* expression in two different types of cancer cells (KU812, a chronic myeloid leukaemia cell line; and DU145, a prostate cancer cell line): silencing SP1 decreased AGAP2 protein levels. We have also found that all-trans retinoic acid (ATRA) treatment increased AGAP2 protein levels in both cell lines whilst curcumin treatment reduced ATRA-mediated AGAP2 increase. Furthermore, chromatin immunoprecipitation studies revealed the presence of RARα, RXRα and the lysine acetyl transferase PCAF in *AGAP2* promoter. Our results provide a novel understanding of *AGAP2* expression regulation that could be beneficial to those patients with cancers where AGAP2 is overexpressed.

## Introduction

AGAP2 (Arf GAP with GTP-binding protein-like domain, Ankyrin repeat and PH domain 2) is a protein that belongs to the Arf GAP (Arf GTPase activating protein) family of proteins. Amongst other functions, the proteins in this family act as GTPase switches for Arfs (ADP ribosylation factors), which are proteins that belong to the Ras superfamily of guanine nucleotide binding proteins. As such, Arf GAPs are involved in signalling regulation. In AGAP2 case, this regulation has been linked to the activity of: AKT, with AGAP2 binding and stabilising AKT in its active conformation^1^; NFκB^2^, with phosphorylated AGAP2 increasing significantly NFκB-mediated transcriptional activity; p53^3^, with AGAP2 increasing its degradation; AMPK^4^, where Fyn-phosphorylated AGAP2 binds to this AMPK and leads to a repression in its signalling pathway; FAK^5,6^; CDK5^7^, with AGAP2 being phosphorylated by CDK5 and leading to an accumulation of activated AKT in the nucleus of postmitotic neurons; and STAT5a, with AGAP2 associating directly with STAT5a and promoting its interaction with the prolactin receptor^8^. The variety of proteins that bind to or are regulated by AGAP2 account for this protein role in cell survival, apoptosis^9^, migration and lipid metabolism^10^ so far.

Whilst there is growing evidence for the role of AGAP2, there is little information available about the gene and no information about its expression regulation. There are two human gene isoforms for *AGAP2*: *AGAP2 isoform 1*, coding for PIKE-L and *AGAP2 isoform 2*, coding for AGAP2 and known previously as PIKE-A or centaurin gamma 1^11^. These two gene isoforms share most of their DNA sequence, with only the sequence of the first exon and first intron differentiating them: the first exon for AGAP2 is located upstream of the first exon for PIKE-L and its expression is controlled by an alternative promoter, making PIKE-L and AGAP2 identical except in their N-terminus. PIKE-L seems to be brain-specific^12^ and its tissue-restriction distribution could be achieved by hypermethylation of the CpG island located on its promoter/first exon region (mapped as part of the ENCODE project). However, AGAP2 expression is considered ubiquitous and, furthermore, its levels are increased in several cancers (prostate cancer, glioblastoma and other tumours) and are associated to tumour progression^13^.

It has been suggested that AGAP2 overexpression is, in some cases, linked to the amplification of the *CDK4* amplicon that occurs in several cancers^14^: *AGAP2* gene is located in chromosome 12 adjunct to the *CDK4* gene and, recently, it has been established that AGAP2 and CDK4 increased co-expression drives glioblastoma progression^15^. However, AGAP2 overexpression is not always due to a duplicon. Therefore, a better understanding of *AGAP2* expression regulation could support a more specific treatment for patients. In this study we have cloned *AGAP2* isoform 2 proximal promoter from genomic DNA, and studied the regions that were contributing to *AGAP2* expression using prostate cancer cell lines and chronic myeloid leukaemia (CML) cell lines as models. Whilst AGAP2 expression in prostate cancer was reported before, this is the first study that links AGAP2 to CML. Furthermore, we also demonstrate a novel role for SP1 and ATRA on AGAP2 transcription activation.

## Results and Discussion

### *AGAP2* expression in chronic myeloid leukaemia

**Figure 1:**
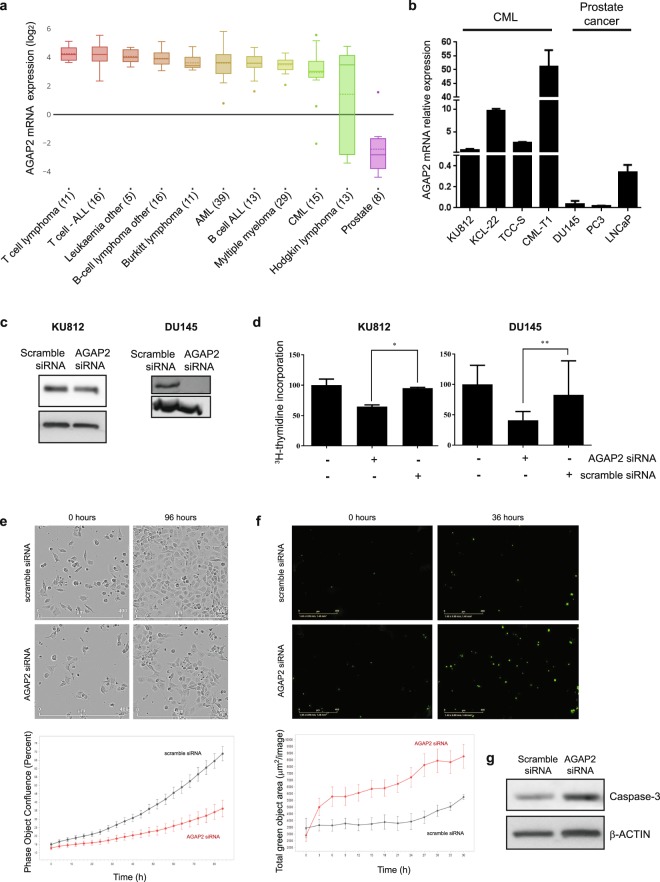
AGAP2 expression and involvement in proliferation in CML and prostate cancer cell lines. (**a**) *AGAP2* mRNA expression comparison between prostate cancer cell lines and blood-related malignancies cell lines. Box-and-whisker plots show the distribution of *AGAP2* expression for each subtype and the dotted line represents the mean. Number of cell lines analysed are indicated in parentheses. Data and customised plot were obtained from the Cancer Cell Line Encyclopedia (CCLE). (**b**) *AGAP2* mRNA basal levels were measured by RT-qPCR in chronic myeloid leukaemia cell lines (CML) KU812, KCL, TCC-S, CML-T1 and in prostate cancer cell lines DU145, PC3 and LNCaP. The values presented were normalised against the levels for the housekeeping genes *TBP* and *HPRT*. (**c**) Cells were transfected with either scramble siRNA or *AGAP2* siRNA for 48 h (KU812, 67 nM siRNA) or 96 h (DU145, 5 nM siRNA). The effect of AGAP2 knockdown on KU812 and DU145 cells proliferation was assessed using [^3^H] thymidine incorporation assays or real-time cell count. (**d**) [^3^H] thymidine was added to the cells during the last 24 h of incubation and the graph represents the averages of five independent replicates. The data were made relative to the values in scramble-transfected cells (which were given a value of 100). (**e**) Cells were rested for 15 min after transfection and then transferred into the IncuCyte® system to be scanned every 4 h for real-time cell count. Cropped images showing cell proliferation are presented in the upper panel (full images can be found in Supplementary Fig. S1). Below, the percentage of phase object confluence of three replicates is shown. (**f**) Silencing AGAP2 induces apoptosis in DU145 cells. DU145 cells were transfected with 5 nM scramble siRNA or *AGAP2* siRNA and after 36 h incubation, cells expressing Annexin V on their surface were detected with the IncuCyte® S3 Live-Cell Analysis system from Essen Bioscience (Ann Arbor) (green mask shown as green dots in the images and total green area (µm^2^/image) over time was quantified in the graph below). (**g**) Levels of active caspase-3 and β-actin were detected by immunoblotting in DU145 cells transfected with either scramble siRNA or *AGAP2* siRNA (5 nM). Error bars represent S.D.;*P < 0.05; **P < 0.01.

AGAP2 protein overexpression is well characterised in prostate cancer^16^. However, although *AGAP2* mRNA has been found in human peripheral blood lymphocytes^17^ and human polymorphonuclear neutrophils^18^, there are no reports to date studying AGAP2 expression and its role in chronic myeloid leukaemia (CML). Interestingly, the Cancer Cell Line Encyclopedia (CCLE) analysed sequencing data from at least 947 human cancer cell lines^19^ and the *AGAP2* mRNA levels found in blood-related malignancies, including CML, is high when compared to the levels found in prostate cancer cell lines (Fig. 1a). Here, we have analysed *AGAP2* mRNA levels in the CML cell lines KU812, KCL-22, TCC-S and CML-T1 as well as in the prostate cancer cell lines DU145, PC3 and LNCaP (Fig. 1b). We observed a clear difference on *AGAP2* mRNA levels between the two cancer types matching the RNAseq findings from the CCLE. To investigate if AGAP2 has a role in CML proliferation, as it has been described for prostate cancer cells^16^, we selected the cell line KU812 as representative for CML. Whilst CML-T1 showed very high levels of *AGAP2* mRNA, KU812 contained levels similar to those found in KCL-22 and TCC-S and it had the advantage of being commercially available. On the other hand, PC3 and LNCaP cells do not express PTEN protein^20^. As PTEN is a regulator of AKT activity and there is a cross-talk between AGAP2 and AKT, PTEN downregulation could have an effect on normal AGAP2 expression regulation. Therefore, we used the prostate cancer cell line DU145 as a comparison to study AGAP2 expression.

It has already been established that AGAP2 expression is required for prostate cancer proliferation^2^. In order to study AGAP2 involvement in CML proliferation, ^3^H-thymidine incorporation assays and real-time cell counts were performed in KU812 and DU145 cells transfected with *AGAP2* siRNA or scramble siRNA. KU812 cells required a higher concentration of *AGAP2* siRNA (67 nM) to achieve a not as strong reduction in AGAP2 protein levels (Fig. 1c). The differences on siRNA concentrations required could be related to CML cell lines being notoriously difficult to transfect, or to the fact that *AGAP2* mRNA levels were considerably higher in KU812 than those found in DU145 (Fig. 1a). AGAP2 knock down significantly decreased ^3^H-thymidine incorporation in both cell lines (Fig. 1d). However, the decrease was more evident and more significant in DU145 cells (where AGAP2 knock down was more efficient). This differential effect was mimicked in the real-time cell count outcome (Fig. 1e and Supplementary Fig. S1). Indeed, we found that AGAP2 silencing was increasing apoptosis in DU145 cells, measured as the amount of cells with Annexin V present in their membrane (Fig. 1f) and the presence of active caspase-3 (Fig. 1g).

CML cells proliferation relies on BCR-ABL1 activation of PI3K/AKT signalling pathway, often through direct activation of PI3K or AKT. But BCR-ABL1 also activate RAS signalling pathways^21^ to promote survival and this could explain why silencing AGAP2 does not seem to affect KU812 cells in the same manner as DU145 cells. Overall, our results showed that AGAP2 is expressed in both cell types and therefore, can be used as a tool to understand *AGAP2* promoter regulation.

### Cloning *AGAP2* promoter region

There are two broad strategies to minimise AGAP2 role in cancer: (1) to reduce AGAP2 activity by targeting its active domains and/or the proteins it interacts with, or (2) to reduce AGAP2 activity by reducing the amount of protein present. Whilst AGAP2 signalling pathway involved in maintaining proliferation and cell survival has been studied elsewhere, there is little information about how *AGAP2* expression can be altered other than the indication that *AGAP2* is co-amplified with CDK4 in an amplicon found in several cancers^9,22^ where the increase in gene numbers leads to an overexpression of the protein. However, how this protein expression is induced (or repressed) is still unknown. Therefore, to understand *AGAP2* expression regulation we questioned the role of functional elements within its promoter.

**Figure 2:**
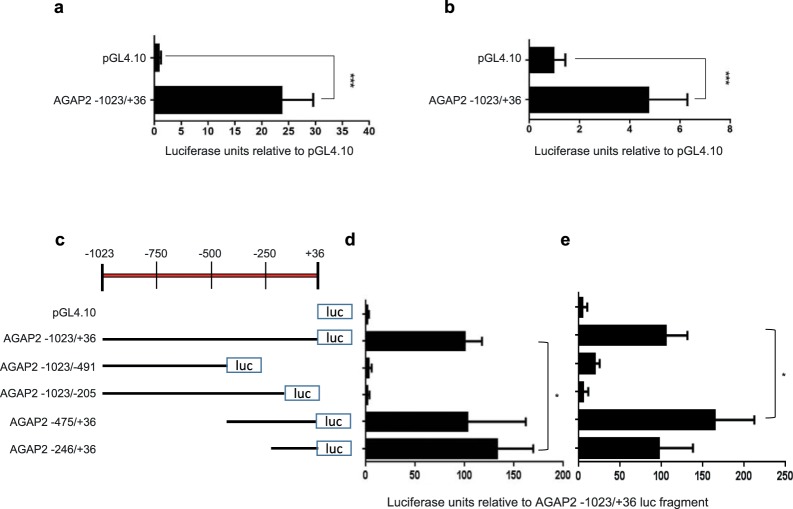
*AGAP2* promoter activity. (**a**) Relative Luciferase activity obtained for the full length AGAP2 −1023/+36 fragment introduced in pGL4.10 and for the empty promoter-less pGL4.10 plasmid when transfected into KU812 cells or (**b**) DU145 cells. (**c**) Schematic representation of the *AGAP2* promoter deletion fragments cloned into pGL4.10 and its luciferase activity in (**d**) KU812 cells and (**e**) DU145 cells. Data throughout the figure are presented as the ratio of the luciferase activity values over the respective β-galactosidase activity and made relative to the values obtained for the full length AGAP2 −1023/+36 plasmid, which received the arbitrary value of 1 (**a** and **b**) or 100 (**d** and **e**). The data shown are the mean ± SD of three independent experiments (n = 8). A Kruskal-Wallis test was performed to confirm differences between the different constructs and comparisons between the full length promoter and one other construct were carried out with a Mann-Whitney U test. (*P < 0.05; ***P < 0.001).

A fragment of ~1000 bp upstream the transcription start site (+1) for *AGAP2* was cloned into the promoter-less luciferase vector pGL4.10 (Promega) and the promoter activity of the fragment was studied with reporter assays. We found significant luciferase activity in this fragment in both cell lines, representative of an active promoter. But the activity was stronger in the KU812 cells (Fig. 2a) compared to the DU145 cells (Fig. 2b). The promoter activity found mimics the levels of *AGAP2* mRNA present in these cells: KU812 cells presented a stronger promoter activity and they also showed a higher levels of mRNA (Fig. 1b).

In order to determine the minimal promoter region and to study functional sequences involved in *AGAP2* expression, several deletion mutants were generated and their reporter activity assayed in both cell lines (Fig. 2c–e). Interestingly, the mutants behaved slightly differently in the two cell lines tested, pointing towards possible cell type-dependent regulatory strategies. In the KU812 cells, the smallest fragment prepared (−246/+36) increased the level of reporter activity found in the original −1023/+36 fragment (Fig. 2d) whilst in the DU145 cell line (Fig. 2e), it was the −475/+36 fragment which presented significantly higher activity than the original −1023/+36 full-length fragment. Overall, the DNA sequence contained in the −246/+36 mutant was sufficient to induce reporter activity in both cell lines, whilst the DNA sequence contained in the −475/−246 fragment seemed to contain important regulatory elements for *AGAP2* expression mainly in the DU145 cell line.

### Analysis of the −246/+36 fragment reporter activity

**Figure 3:**
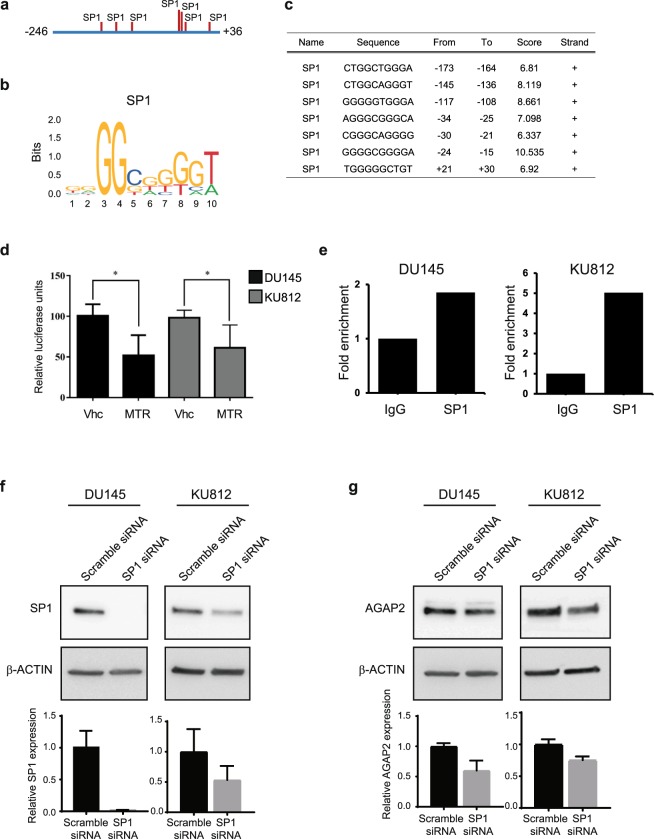
SP1 role in AGAP2 expression. The −246 to + 36 region of *AGAP2* was analysed for SP1 binding sites with the online JASPAR prediction tool. (**a**) Representation of the SP1 binding sites location within the *AGAP2* −246/+36 DNA fragment. The actual logo used for the searches is shown in (**b**) and the scores for binding are provided as a table in (**c**). (**d**) Effect of mithramycin (MTR) treatment on luciferase activity. KU812 and DU145 were transfected with the *AGAP2* −246/+36 luc and the β-galactosidase plasmids and the reporter activity was measured after 24 h in the presence of 200 nM MTR or just vehicle (Vhc). Represented in the graph are the mean ± SD of data from at least three independent experiments performed in triplicates (n = 9). Values were calculated as the ratio of the luciferase activity values over the respective β-galactosidase activity and made relative to the values obtained for Vhc treated cells (control). Treatment differences were analysed with Mann-Whitney U tests (*P < 0.05). (**e**) DU145 and KU812 cells were grown to 80% confluency under normal serum conditions. Then cells were fixed and sheared using the AFA Focused-Ultrasonicator from Covaris and chromatin was immunoprecipitated with 1 μg of rabbit IgG or 1 μg of anti-SP1 antibody (D4C3, Cell Signalling). *AGAP2* promoter region was identified in the immunoprecipitated material using the primers found in Supplementary Table 1. Values are represented with the fold enrichment method (signal over background, using the IgG Ct values as the background). (**f**,**g**) KU812 and DU145 cells were transfected with either scramble or *SP1* siRNA. 48 h after transfection (KU812, 67 nM siRNA) or 72 h after transfection (DU145, 5 nM siRNA), cells were lysed and 10 μg of total protein were used to detect SP1 levels (**f**) and AGAP2 levels (**g**) by western-blotting followed by immune-blotting with specific antibodies. Levels of β-Actin were used as loading control. Densitometry values for the relative protein expression (average ± SD from independent experiments) are represented below the blots in (**f**) and (**g**). Cropped blots are shown and full blots can be found in Supplementary Figures S3 and S4.

*In silico* analysis of the −246/+36 DNA sequence revealed the presence of several putative SP1 binding sites (Fig. 3a,c). SP1 is an abundant transcription factor often involved in constitutive gene expression. To study the relevance of these sites, KU812 and DU145 cells were transfected with the promoter-less plasmid pGL4.10 or AGAP2 −246/+36 luc construct, and their reporter activity was assessed in the presence/absence of the anticancer drug mithramycin. This drug acts as a competitive inhibitor for SP1 binding to GC-rich DNA motifs, causing a global displacement of SP1 transcription factors^23,24^. In both cell lines, the reporter activity was significantly decreased in the presence of mithramycin (Fig. 3d), suggesting the requirement for SP1 binding for an active *AGAP2* promoter. However, as mithramycin has also been reported to reduce RNA synthesis^25^, to rule out the possibility that the reduction in the luciferase activity were due to RNA synthesis inhibition by a mechanism independent of SP1 binding displacement, we mutated one of the SP1 binding sites in the AGAP2 −246/+36 luc construct. As seen in Supplementary Fig. S2, the mutation of a putative SP1 binding site caused a significant reduction of the luciferase activity in DU145 cells, providing support for a SP1 role in *AGAP2* expression. To confirm the presence of SP1 in the *AGAP2* promoter, chromatin immunoprecipitation (ChIP) studies were performed in KU812 and DU145 cells. Under conditions already tested for *AGAP2* expression (cells grown in the presence of serum), SP1 was found bound to AGAP2 promoter (Fig. 3e).

Next, SP1 levels were knocked down with a specific *SP1* siRNA (s13319) and observed the effect on AGAP2 protein in KU812 and DU145 cells. Transfection with *SP1* siRNA reduced SP1 protein levels at 48 h (KU812) and 72 h (DU145) (Fig. 3f). Knocking down SP1 levels did have an impact on AGAP2: the reduction in SP1 was associated with a reduced expression of AGAP2 protein in both cell lines (Fig. 3g). However, this reduction was more prominent in KU812 cells. This could be related to the higher SP1 enrichment found on *AGAP2* promoter in KU812 cells (Fig. 3e, right). Altogether, our results support involvement of SP1 in *AGAP2* expression on the two cell lines studied. Furthermore, taking into account that AGAP2 has been described as a proto-oncogene^14^, these results support previous reports that propose SP1 as a target for chemotherapy^26^.

### Relevant binding sites in the −475/−246 *AGAP2* promoter region

**Figure 4:**
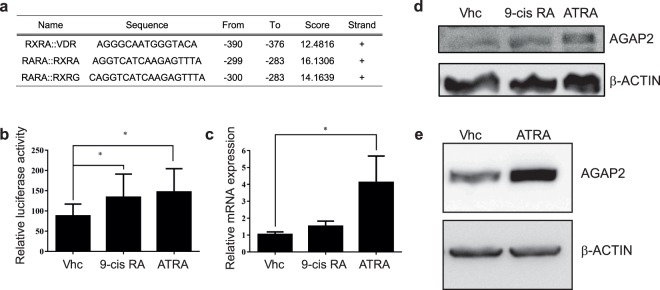
Involvement of retinoid receptors on AGAP2 expression. The −475/−246 region of *AGAP2* promoter was analysed with the JASPAR online database and several binding sites for retinoid-related receptors were identified and are presented as a table in (**a**). To identify any involvement of the retinoid receptors on *AGAP2* expression, DU145 and KU812 cells were grown in clear media with 5% charcoal-stripped serum for the following experiments. (**b**) DU145 cells were transfected with AGAP2 −475/+36 luc plasmid and pCH110 and luciferase and β-galactosidase activities were measured after 24 h treatment with just vehicle (Vhc), 1 μM 9-*cis* RA or 1 μM ATRA. Luciferase values were normalised to the respective β-galactosidase activity values. The normalised luciferase values are expressed as a percentage expression of the untreated (Vhc) AGAP2 −475/+36 luc fragment. (**c**) Changes in *AGAP2* mRNA expression in DU145 cells following 24 h treatment with Vhc, 1 μM 9-*cis* RA or 1 μM ATRA, were measured by RT-qPCR with specific primers, using *TBP* and *RPS18* expression for normalisation. Results shown represent the mean ± SD of data from three independent experiments. AGAP2 protein levels were detected after 24 h treatment with 1 μM ATRA and 9-*cis* RA in DU145 cells (**d**) or after 24 h with 1 μM ATRA in KU812 cells (**e**). The same blots were used to detect β-Actin levels, as loading control. Full immunoblots are shown in Supplementary Fig. S4. Differences between treatments were analysed in (**b**) and (**c**) with a Kruskal-Wallis test, followed by comparisons between two groups with a Mann-Whitney U test. (*P < 0.05).

The AGAP2 −475/+36 luc construct contains about 200 bp more (upstream) than the AGAP2 −246/+36 luc construct. In the DU145 cell line, the presence of those extra 200 bp (the region corresponding to −475/−246 bp), significantly increased the basal luciferase activity contained in the full-length fragment (Fig. 2e). Further *in silico* analysis of the −475/−246 *AGAP2* fragment using the JASPAR database, indicated a putative DR5 binding site (a retinoic acid response element (RARE)^27^) in this region (Fig. 4a). The RAR/RXR heterodimer binds to this element and responds to its ligands 9-*cis* retinoic acid (9-cis RA) and all-*trans*-retinoic acid (ATRA)^28^. In the absence of ligand, the heterodimer is associated with co-repressors, such as N-CoR and SMRT, which recruit or are part of complexes with histone deacetylase activity (HDAC), negatively regulating transcription. In the presence of the ligand, the co-repressor complex dissociates and co-activator complexes with histone acetyl transferases (HATs) are recruited to the promoter activating transcription^29^. Contrary to DU145 cells, in KU812 cells the AGAP2 −475/+36 luc construct showed similar luciferase activity to the shorter −246/+36 fragment. If the different cell line behaviour is indeed mediated through the RARE, it could be explained if RARα levels were lower in KU812 cells, as abnormalities on this gene are common in myeloid disorders^30^ with the gene often downregulated^31^. To test if the RARE was functional and responsible for the increased luciferase activity observed in the DU145 cell line, cells were transfected with the −475/+36 AGAP2-luc fragment in clear, 5% charcoal-stripped medium, and treated with the standard concentration^32^ of 1 μM 9-cis RA or 1 μM ATRA for 24 h. The luciferase activity was significantly increased in the presence of the ligands (Fig. 4b). Furthermore, ATRA treatment also induced a significant increase in *AGAP2* mRNA levels (measured by RT-qPCR, Fig. 4c), that translated into a modest increase in AGAP2 protein (Fig. 4d). Although KU812 cells did not show much difference in the reporter activity of fragment −246/+36 compared to fragment −475/+36, we tested if the DR5 binding site was also functional in these cells by growing them in clear RPMI media with 5% charcoal-stripped serum and treating them with 1 μM ATRA for 24 h. To our surprise, we found the treatment was able to induce AGAP2 protein expression (Fig. 4e). These results demonstrate for the first time that ATRA can mediate *AGAP2* expression in different cancer cell lines. For further confirmation of ATRA involvement, we also demonstrated the presence of the nuclear receptors RARα and RXRα on *AGAP2* promoter by ChIP (see next section).

The overall effect of ATRA in DU145 cells varies in the literature: treatment with 1 μM ATRA has been reported to induce from cell cycle arrest at 24 h^33^ to either apoptosis^34^ or moderate growth arrest at 72 h^35^. In our hands, prolonged ATRA treatment also lead to DU145 cell death (data not shown). Therefore, we were presented with the paradox that while DU145 cells required AGAP2 function for cell survival (Fig. 1) and ATRA was an effective inducer of AGAP2 expression (Fig. 4), longer exposure to ATRA was counteracting for cell growth.

The different effects of ATRA treatment can be better understood when considering the work of Hammond *et al*.^36^. They showed that RAR agonists and RAR antagonists affected prostate cancer cell lines, suggesting that cell survival might depend on a balance of RAR-mediated transcriptional activity. But crucially, they also showed that a component in serum could diminish the effect of some of these compounds. Indeed, the IC50 value for ATRA in DU145 cells has been described to be 135 μM in the presence of 10% serum^37^, which is far higher than the 1 μM concentration used in this study and in those showing cell arrest at 24 h^33^ or apoptosis at 72 h^34^. But in these last two studies, DU145 cells were grown in serum-free medium 24 h prior ATRA treatment and during the duration of the treatment. And, in our study, DU145 cells were grown in 5% charcoal-stripped serum-containing medium. Therefore, the absence of serum seems to exacerbate ATRA anti-proliferative effects.

### Involvement of specific lysine acetyl transferases on *AGAP2* expression

**Figure 5:**
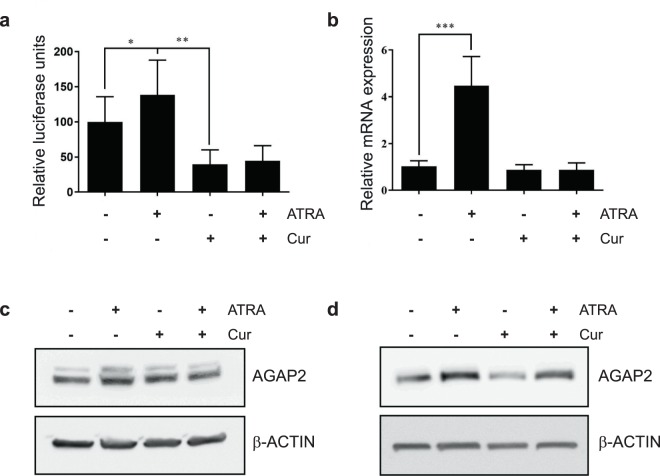
Effect of curcumin on AGAP2 expression in DU145 cells. (**a**) Relative luciferase values corresponding to cells transfected with the AGAP2 −475/+36 luc plasmid and treated with 10 µM curcumin for 1 hour, followed by 24 h incubation with the RAR-α ligand ATRA. The luciferase activity was normalised to the corresponding β-galactosidase values and the data were expressed as a percentage of the untreated control cells. (**b**) AGAP2 mRNA expression in cells treated with either 1 μM ATRA for 24 h, 10 μM curcumin for 24 h, or pre-treated with 10 μM curcumin for 1 h followed by the administration of 1 μM ATRA for 24 h. AGAP2 mRNA expression values were normalised to *TBP* and *RPS18* expression. The values were expressed in relation to the untreated control cells. The data are represented as means ± SD and correspond to three experiments performed in triplicates (n = 9). Differences between the treatments in (a) and (b) were analysed using a Kruskal-Wallis test followed by comparisons between two groups analysed with a Mann-Whitney U test (*P < 0.05; **P < 0.01; ***P < 0.005). (**c**) DU145 cells and (**d**) KU812 cells were grown overnight in clear medium with 5% charcoal-stripped serum. They were then treated for 24 h with either 1 μM ATRA or 10 μM curcumin or pre-treated with 10 μM curcumin for 1 h and then treated with 1 μM ATRA for 24 h. Cells were then lysed in RIPA buffer and 20 μg of protein were loaded onto a SDS-PAGE, transferred to a nitrocellulose membrane and blotted with specific antibodies for AGAP2 and β-Actin proteins. Full blots are shown in Supplementary Figures S6 (DU145 cells) and S7 (KU812 cells).

Upon ligand binding, the RAR/RXR heterodimer recruits coactivator complexes to the gene promoter area. These complexes contain several proteins, some of them with histone acetyl transferase activity (HATs). HATs were renamed in 2007 as lysine acetyl transferases (KATs), and the 17 members in the human KAT superfamily were divided in families based in their transferase domain sequence conservation^38^. Within these families, we find CBP (KAT3A)/p300 (KAT3B) family and GCN5 (KAT2A)/PCAF (KAT2B) family. Both of them have been associated with ATRA-mediated transcriptional activity^39–41^. To study if ATRA-mediated activation of *AGAP2* promoter involved any of these KATs, DU145 cells were pre-treated with curcumin. Curcumin has been widely used as a general KAT inhibitor, targeting the KATs p300 and CBP, Tip60 and PCAF^42^. Cells were transfected with the −475/+36 AGAP2-luc plasmid and treated with either 1 μM ATRA or 10 μM curcumin (10 μM and 20 μM curcumin were found to have the same effects in preliminary experiments), or with 10 μM curcumin followed by 1 μM ATRA administration 1 h later. After 24 h treatment, ATRA-mediated increase in luciferase activity was completely abolished in the presence of curcumin (Fig. 5a). Seemingly, ATRA-induced increase in *AGAP2* mRNA levels was returned to basal levels (Fig. 5b) and protein levels were further reduced (Fig. 5c,d with full blots shown in Supplementary Figs S6 and S7) in the presence of 10 μM curcumin. Therefore, curcumin pre-treatment managed to counteract ATRA-mediated increase in AGAP2 levels. These results support the involvement of KATs in ATRA-mediated effect. However, curcumin is a promiscuous binder that can affect the activity of proteins other than KATs^42^. Indeed, curcumin has been found to decrease the expression of SP1 in bladder cancer cells^43^ and consequently these results are not conclusive of involvement of KATs in ATRA-mediated effect but they do show that curcumin is an efficient agent to reduce ATRA-mediated AGAP2 expression in both DU145 and KU812 cells.

**Figure 6:**
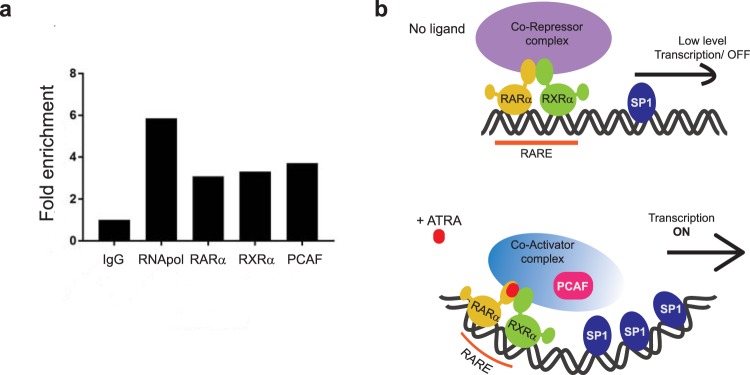
Mechanism of ATRA-mediated AGAP2 transcription. (**a**) Sheared chromatin of DU145 cells grown under AGAP2 expression conditions was used for immunoprecipitation using 2 μg of antibody (a rabbit IgG as negative control, a rabbit antibody against RNApol II as positive control, a rabbit anti-RARα, a rabbit anti-RXRα or a rabbit anti-PCAF antibody) and optimised primers were used to amplify a region specific to the *AGAP2* promoter (see Supplementary Table 1). Data are represented as fold enrichment: fold enrich in signal relative to the IgG background signal. (**b**) Diagram representing the proposed mechanism of ATRA-mediated activation of *AGAP2* transcription. In the presence of ATRA (lower panel), the heterodimer RARα/RXRα would recruit the lysine acetyl transferase PCAF to activate transcription and the recruitment of SP1 would also be enhanced.

In order to identify possible KATs involved in AGAP2 expression, we used another approach: we investigated the enrichment of candidate KATs on *AGAP2* gene promoter. A preliminary co-immunoprecipitation study determined which KATs formed complexes with RARα in DU145 cells (see Supplementary Fig. S8). Specific antibodies against CBP, PCAF or GCN5L2 were used and we found that both the co-activator PCAF and the lysine acetyl transferase GCN5L2, which belong to the KAT2 family, were present in the co-precipitated fraction under normal growth conditions (media with serum). Next, chromatin immunoprecipitation studies were performed in the presence of serum in DU145 cells and confirmed that RARα, RXRα and PCAF were all found in *AGAP2* promoter (Fig. 6a). These findings provide a possible mechanism for *AGAP2* regulation, where, in the presence of serum (low level of natural occurring retinoids) or upon the addition of ATRA, the RAR/RXR heterodimer would mediate *AGAP2* expression by recruiting the acetyl transferase PCAF to the promoter.

That ATRA and SP1 both mediate AGAP2 expression provides an interesting possibility. There are several reports that describe enhanced SP1 binding to promoter regions after ATRA treatment^44,45^, even with the possibility of RARα recruitment to promoters without RARE through direct interaction with SP1^46,47^. Furthermore, the acetyl transferase activity of PCAF, associated to ATRA transcription mediation^41^, is necessary to stimulate SP1-driven transcription^48^. We therefore propose a mechanism for AGAP2 expression where presence of ATRA would lead to PCAF recruitment and increased SP1 binding to the promoter region (Fig. 6b).

Overall, we have shown for the first time that AGAP2 expression is mediated by SP1 and ATRA in KU812 and DU145 cells and that curcumin treatment reduces ATRA-mediated expression of AGAP2. We have also demonstrated that the RARα/RXRα heterodimer and the KAT PCAF are found in *AGAP2* promoter under conditions of active *AGAP2* expression. Our results provide a novel understanding of *AGAP2* expression regulation that could be beneficial to those patients with cancers where AGAP2 is overexpressed.

## Methods

### Cell culture and reagents

Chronic myeloid leukaemia (CML) human-derived cell lines KU812 and TCC-S were obtained, respectively, from the American Type Culture Collection (ATCC) repository and Dr Yuko Sato^49^ via Dr Felipe Prosper. KU812 and TCC-S were maintained in RPMI media supplemented with 10% FBS (Gibco), 1% L-Glutamine and 10 mM HEPES buffer. Human prostate carcinoma DU145 cells were obtained from the ATCC and cultured in DMEM supplemented with 10% FBS (Gibco) and 1% L-Glutamine. Frozen cells pellets for the CML cell lines KCL-22 and CML-T1 as well as cell pellets from the prostate carcinoma cell lines PC3 and LNCaP were a gift from the John van Geest Cancer Research Centre. They obtained the PC3 and LNCaP cells from the ATCC, the CML-T1 cells from the Leibniz Institute DSMZ and the KCL-22 cells from Antony Nolan via Prof. Tony Dodi. When treatments involved the retinoid receptors, the cells were washed with PBS and grown in clear media supplemented with 5% charcoal-stripped FBS, 1% L-Glutamine (Lonza), and penicillin/streptomycin. All cell lines were maintained in a 37 °C, 5% CO_2_ fully humidified incubator. The authenticity of the two main cell lines used in this study was analysed and confirmed by the European Collection of Authenticated Cell Cultures after all experiments were completed.

*AGAP2* siRNA and the *SP1* siRNA (ID s13319) were purchased from Ambion/ThermoFisher, and the scrambled siRNA from Santa Cruz Biotechnology. Mithramycin was purchased from Sigma, dissolved in methanol and used for 24 h treatments at 200 nM.

### Gene expression

Total RNA was extracted from cell pellets using a total mammalian RNA extraction kit (Sigma). The RNA was quantified using Nanodrop800 spectrophotometer (Fisher Scientific) and reverse transcribed into cDNA using the M-MLV Reverse Transcriptase (Promega). *AGAP2* expression was detected using primers designed with Primer3 (AGAP2 fwd 5′-CTCAGGAAGCTGGCAGAGAG-3′, AGAP2 rev 5′-AGCGGCTCAAAGTCCATTC-3′) in a Corbett Research PCR machine (Qiagen), following the instructions of the iTaq Universal SYBR Green Supermix (Bio-Rad). Product amplification was analysed using the 2^ΔΔCt^ method. *AGAP2* expression was normalised to *HPRT* and *TBP* expression (primer sequences in Supplementary Table 1). When retinoic acid treatments were provided, *AGAP2* expression was normalised to *TBP* and *RPS18* expression, with the latter detected using Bio-Rad PrimePCR™ SYBR® Green Assay: *RPS18*, Human.

### *AGAP2* silencing and proliferation studies

KU812 cells were transfected with 67 nM *AGAP2* siRNA or 67 nM scramble siRNA via electroporation (Amaxa nucleofection V kit, Lonza) in accordance with the manufacturer’s instructions and using the program x-001 in the Nucleofector II Amaxa Biosystems (Lonza). DU145 cells were seeded in 96 well plate at 5 × 10^3^ cell per well, and transfected with 5 nM *AGAP2* siRNA or 5 nM scramble siRNA using Interferin (Poliplus) in accordance with the manufacturer’s protocol. The transfection was carried out for 48 h (KU812 cells) or 96 h (DU145 cells) and ^3^H-thymidine was added to each well 24 h before the end of the incubation time to a final concentration of 0.037 MSq/ml. The cells were harvested and the incorporated ^3^H-thymidine quantified using a TopCount NXT microplate scintillation counter. Transfections were made in parallel to extract the total protein and confirm that AGAP2 levels were knocked down by *AGAP2* siRNA. The process of protein extraction and AGAP2 detection is described under the ‘Immunoprecipitation and western blotting’ heading below. Real-time proliferation was also analysed with the IncuCyte® S3 Live-Cell Analysis system from Essen Bioscience (Ann Arbor). Cells were transfected and seeded in serum-free medium at a density of 6 × 10^3^ and 4 × 10^4^ cells/well for DU145 and KU812, respectively. For KU812 cells, the plate was previously coated with 0.01% poly-L-lysine solution. Following seeding, the cells were allowed to settle for 15 min at room temperature and then transferred to the IncuCyte® system and wells were scanned every 4 h for 48 h (KU812) or 96 h (DU145) using a 10x objective. The results were analysed using the integrated confluence algorithm and presented as percentage of confluence over time.

### Apoptosis detection

The Annexin V reagent (Essen Bioscience) was used to detect apoptosis in real-time. Cells transfected with either scramble siRNA or AGAP2 siRNA (see conditions for DU145 transfection above) were seeded at a density of 3 × 10^3^ cells/well in 200  μl complete media with Annexin V reagent (1:200). The cells were scanned every 3 hours for 36 hours at 10x magnification using a green fluorescence channel. The results were analysed using IncuCyte® Live-Cell analysis software and presented as total green area (μm^2^/image) over time. 96 hours after transfection, the cells were lysed and 10 µg of proteins were used to detect active caspase-3 levels using the caspase-3 (H-277) antibody from Santa Cruz Biotechnology.

### *AGAP2* promoter cloning

PCR primers specific to the *AGAP2* promoter sequence available at the Genome Browser (assembly release date Dec 2013) were designed to generate the −1023/+36, −1023/−205, −1023/−491, −475/+36 and −246/+36 AGAP2-luc constructs (see Supplementary Table 1). Human genomic DNA (Promega) was used as a template. The amplified *AGAP2* promoter fragments were cloned into the promoter-less firefly luciferase vector pGL4.10 (Promega) and the sequences were verified by Sanger sequencing (Source Biosciences). The *in silico* analysis of the sequences were carried out with the online JASPAR database^50^. 10 ng of the plasmid −246/+36 AGAP2-luc were used to mutate a SP1 binding site, following the protocol originally developed by Stratagene and using Pfu DNA polymerase (Life technologies). The reaction was carried out for 16 cycles, with a 55 °C annealing temperature. Primers containing the mutation were designed according to the recommendations in the QuickChangeTM protocol and can be found in the Supplementary Table 1. Digestion of the parental, un-mutated plasmid DNA was performed using the *Dpn*I restriction enzyme (Promega). The incorporation of the mutation was assessed by Sanger sequencing followed by nucleotide alignment to the wild type sequence, performed with the online software MultAlin^51^.

### Transfection and Luciferase Assays

Briefly, 2 × 10^6^ KU812 cells were transfected via electroporation with 1 µg of the different *AGAP2-*luciferase reporter plasmids or pGL4.10, and 1 µg of pCH110 (Amersham). Then cells were transferred into 24 well plates and left for 24 hours. When treated, the cells were allowed to recover for 4 hours and then the treatment was added to the culture medium, for 20 hours. 24 hours post electroporation, the cells were lysed and the luciferase activity analysed with the Dual-Light™ Luciferase & β-Galactosidase Reporter Gene Assay System (ThermoFisher) in accordance with the manufacturer’s instructions. Reporter activity was measured with FLUOstar Optima device (BMG Biotech). The luciferase activity was normalised to the corresponding β-galactosidase values.

2 × 10^4^ DU145 cells were seeded into 24 well plates and transfected with 0.5 µg of the relevant *AGAP2*–luciferase plasmid and 0.5 µg of pCH110 using the CalPhos Mammalian Transfection kit (Clontech). After 12 hours, the growth medium was replaced and the cells were allowed to recover for 24 hours. Treatments were provided at the end of the recovery period for an additional 24 hours and the luciferase activity measured as above. If curcumin was used, the growth media was changed to clear DMEM and a 10 µM curcumin treatment was carried out for 1 hour. Then 1 µM ATRA was added for the indicated times.

### SP1 silencing

KU812 and DU145 cells were transfected with scramble or *SP1* siRNA (s13319) with the same method and concentration described for AGAP2 silencing. The transfection was carried out for 48 h (KU812 cells) or 72 h (DU145 cells). And afterwards, SP1 and AGAP2 protein levels were detected by immunoblotting as described below.

### Immunoprecipitation and western blotting

After relevant treatments, proteins were extracted with RIPA buffer and their concentration measured with the BCA kit (Pierce). For immunoprecipitations, 2 µg of anti-RARα goat antibody (L-15; Santa Cruz Biotechnology) were coupled with the 50 µl of Surebeads protein – G (BioRad) in a total volume of 200 µl of TBST for 10 minutes at room temperature. The beads were washed 3 times with TBST and 500 µg of protein lysate added to the beads in a total volume of 500 µl of RIPA buffer. This mixture was incubated at 4 °C overnight. Following washes with TBST, the antibody-protein complex were eluted from the beads using a 20 nM Glycine (pH 2.0) solution for 5 minutes. The reaction was quenched with 10% (v/v) 1.5 M Tris (pH 7.5) and the supernatant used for western blotting. For direct lysis to western blot, 50 µg of proteins were used (unless otherwise stated). Proteins were resolved on 8–10% SDS-PAGE gels, transferred onto nitrocellulose membranes and probed for AGAP2 (1:2000, PIKE antibody, Sigma), RARα (1:1000, C-20, Santa Cruz Biotechnology), SP1 (1:1000, D4C3, Cell Signalling), the Lysine Acetyltransferase Antibody Sampler Kit (1:1000, Cell Signalling) and β-actin (1:5000, Sigma). Membranes were developed and analysed using the FUJI – LAS 3000 imaging system.

### Chromatin immunoprecipitation

DU145 cells were seeded at 1 × 10^6^ cells per 10-cm dish and grown until they were 80% confluent. KU812 cells were seeded and left overnight. Then, chromatin immunoprecipitation (ChIP) was performed using the truChIP™ Chromatin Shearing Reagent kit from Covaris and MAGnify™ Chromatin Immunoprecipitation System from Invitrogen and following the manufacturer’s instructions. The chromatin was sheared to 100–600 bp by sonication using an AFA Focused-Ultrasonicator (S220- Series from Covaris^TM^) at 6 × 60 second on-off pulses at 4 °C. Afterwards, crosslinked proteins of interest were immunoprecipitated with 2 μg of either anti-RARα (C-20), anti-RXRα (D-20) (both from Santa Cruz Biotechnology), anti-PCAF (C14G9, Cell Signaling), or 1 μg of anti-SP1 (D4C3, Cell Signalling). Anti-Pol II (N-20, Santa Cruz Biotechnology) was used as positive control and rabbit IgG (Invitrogen) as negative control. Immunoprecipitated DNA was purified and used for qPCR amplifications. (Primer sequences for qPCR analysis can be found in Supplementary Table 1).

### Statistical Analysis

Data are presented as mean ± standard deviation (SD). As the data were originated from independent repeats, they were transformed into percentages or ratios before performing statistical analysis. Differences between the means were assessed by non-parametric tests using GraphPad Prism 5 software. Significance level was assigned at *P* < 0.05.

## Supplementary information

Supplementary Information
