# Supplementary material for: SP1 and RARα regulate AGAP2 expression in cancer
Source: Sci Rep. 2019 Jan 23;9:390. doi: 10.1038/s41598-018-36888-x (PMC6344547; doi:10.1038/s41598-018-36888-x)
Supplement: Supplementary file 1 — Supplementary Information [file 41598_2018_36888_MOESM1_ESM.pdf]

Supplementary Information

SP1 and RARα regulate *AGAP2* expression in cancer

Yegor Doush[1], Arif A. Surani[1], Amaia Navarro-Corcuera[1,2], Stephanie McArdle[3], E. Ellen Billett[1] and Cristina Montiel-Duarte*[,1]

[1] College of Science and Technology, Nottingham Trent University, Nottingham, UK
[2] Department of Biochemistry and Genetics, University of Navarra, 31008 Pamplona, Spain
[3] The John van Geest Cancer Research Centre, Nottingham Trent University, Nottingham, UK

* *Corresponding author:* Dr Cristina Montiel-Duarte, School of Science and Technology, Nottingham Trent University, Clifton campus, Nottingham NG11 8NS (UK). Tel: +44 (0) 115 8486646. Email: cristina.montielduarte@ntu.ac.uk

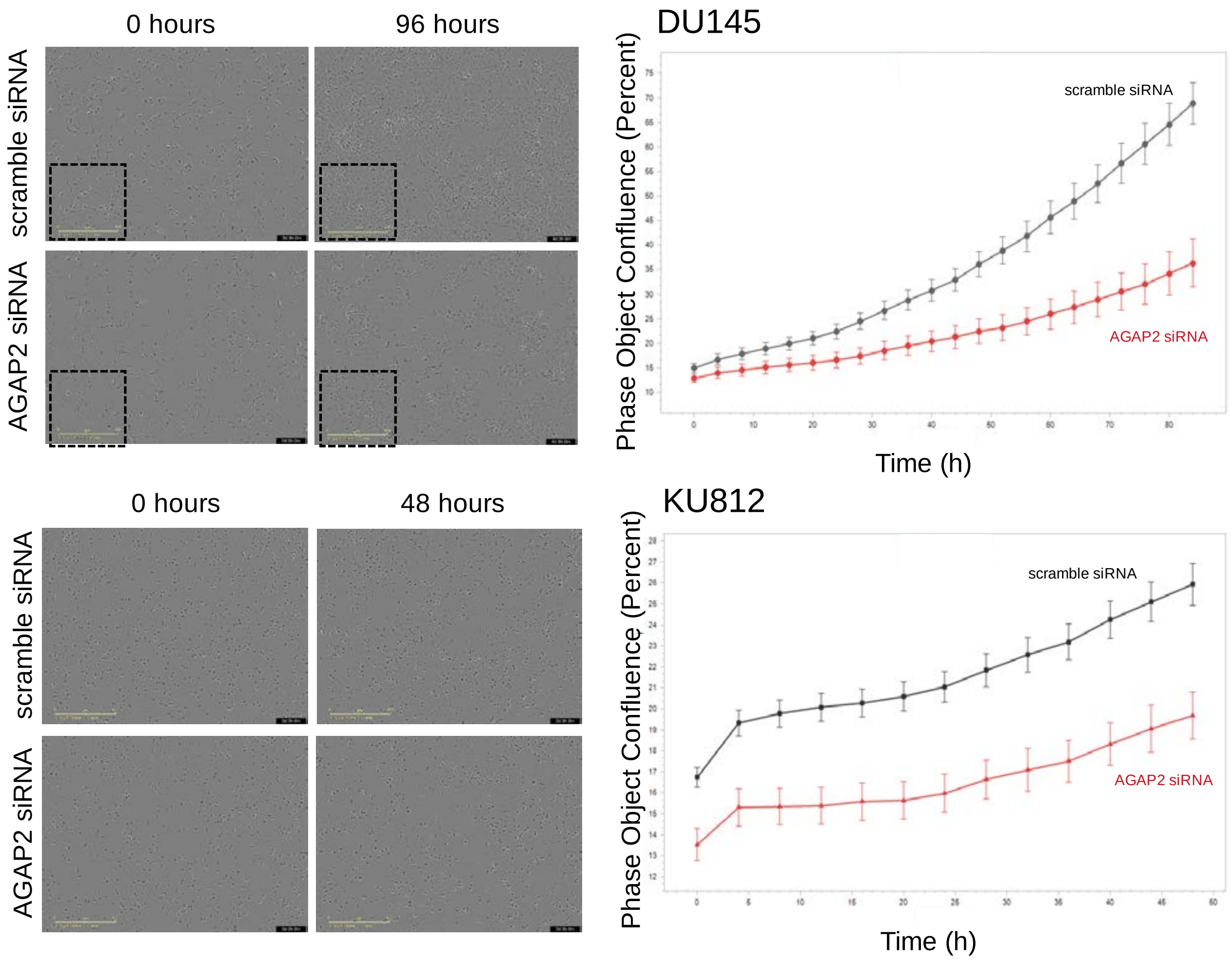

Supplementary Figure S1. **Role of AGAP2 in DU145 and KU812 proliferation**. Cells were transfected with either scramble siRNA or AGAP2 siRNA for 48 h (KU812, 67 nM siRNA) or 96 h (DU145, 5 nM siRNA). Cells were rested for 15 min after transfection and then transferred into the IncuCyte® system to be scanned every 4 h for real-time cell count. The results are presented as representative images of the cultured wells and graphs with the percentage of phase object confluence for each condition. Data represent the mean ± SD of data from three independent replicates (n =3). The dotted square corresponds approximately to the cropped area shown in Figure 1e.

Supplementary Table 1. List of primers used.

	Name	Sequence
Promoter cloning	F (-1023)	5'- GATCCTCGAG AACCAATCTGGGCTTTATGTGCTTGG-3'
	F (-246)	5'- GATCCTCGAG CCACAGGAGGCCCTGGGACA-3'
	F (-475)	5'- GATCCTCGAG CAGGAGGTGGCCTCAGTGGGT-3'
	R (+36)	5'- GATCAGATCT GGCCCTACAGCCCCCAAACCT-3'
	R (-205)	5'- GATCAGATCT GCCTCCTGTGGGAGGTGGCT-3'
	R (-491)	5'- GATCAGATCT CCCACTGAGGCCACCTCCTGT-3'
Mutagenesis	SP1 fwd	5' - CTTCTGAGGTTTGGGG**TA**TGTAGGGCCATGGGCC - 3'
	SP1 rev	5' - GGCCCATGGCCCTACA**TA**CCCCAAACCTCAGAAG - 3'
ChIP	AGAP -218 rev	5' - CAAGCTAGGTCCGAGGTGC - 3'
	AGAP2 -425 fwd	5' - GTGTAGAGAGGGCAATGGGTA C- 3'
qPCR	TBP fwd	5'-TTCGGAGAGTTCTGGGATTG-3'
	TBP rev	5'-GGATTATATTCGGCGTTTCG-3'
	HPRT fwd	5'-ATGCTGAGGATTTGGAAAGG-3'
	HPRT rev	5'-AATCCAGCAGGTCAGCAAAG-3'

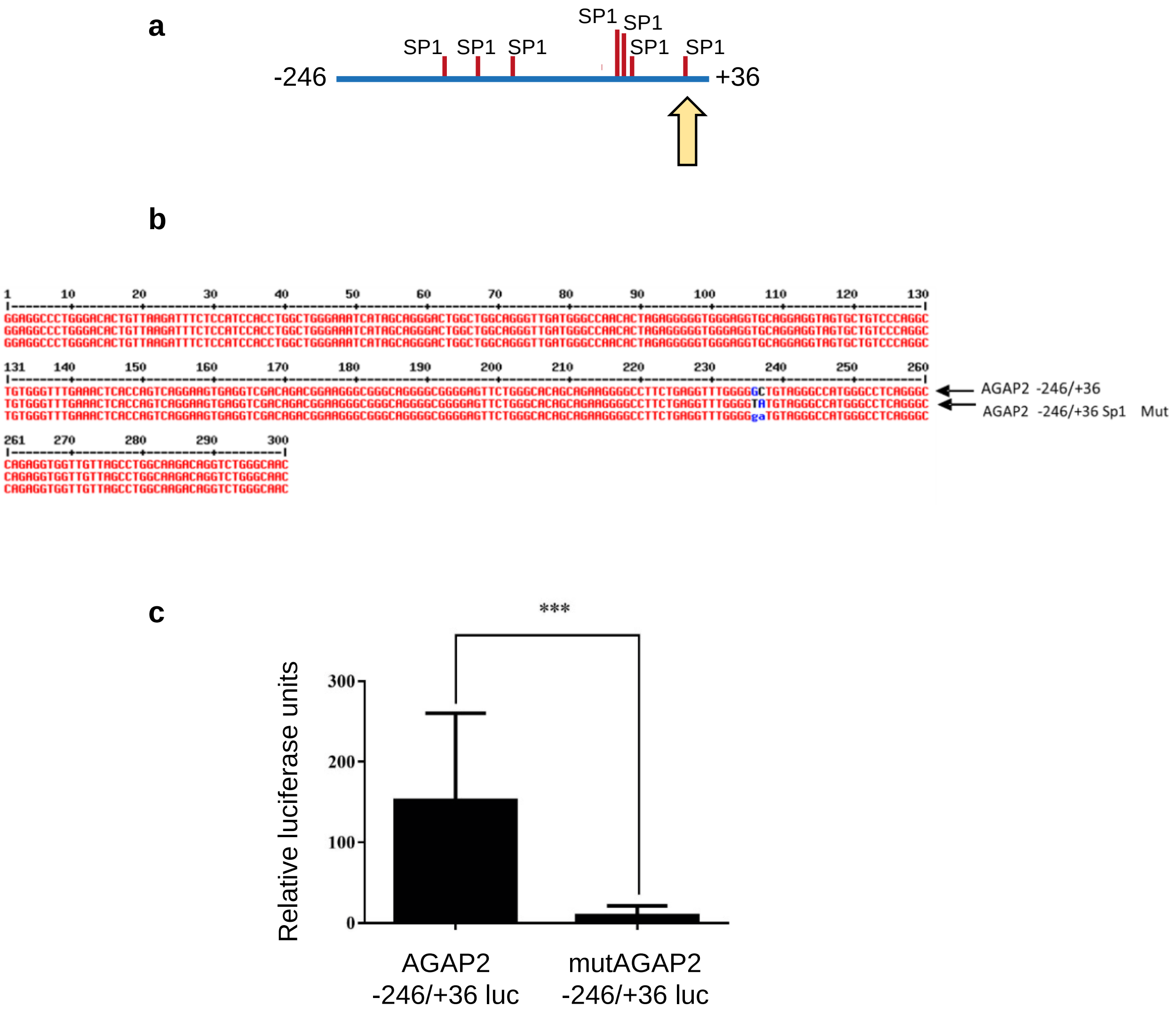

Supplementary Figure S2. **Role of SP1 in *AGAP2* proximal promoter**. There are several putative SP1 binding sites within *AGAP2* proximal promoter **(a)** and the one indicated with the arrow, located a few nucleotides after the transcription start site, was mutated using site-directed mutagenesis (primers available in Supplementary Table 1). **(b)** Nucleotide alignment for AGAP2 wild type sequence and the results of the site-directed mutagenesis confirmed that the plasmid mutAGAP2 -246/+36 luc contained the desired mutation, disrupting the SP1 binding site. The alignment was performed using the on-line software MultAlin. **(c)** The effect of the mutation on AGAP2 expression was analysed with reporter assays. DU145 cells were transfected with the wild type AGAP2 -246/+36 luc plasmid or with a mutated version (mutAGAP2 -246/+36 luc) and the luciferase activity generated by these constructs was measured. Cells were co-transfected with plasmid pCH110 and β-galactosidase activity was measured as an internal control for transfection. Bars represent the mean ± SD of data from at least three independent experiments performed in triplicates (n =9). Differences in the mean were analysed with a Mann-Whitney U test (***$P < 0.001$)

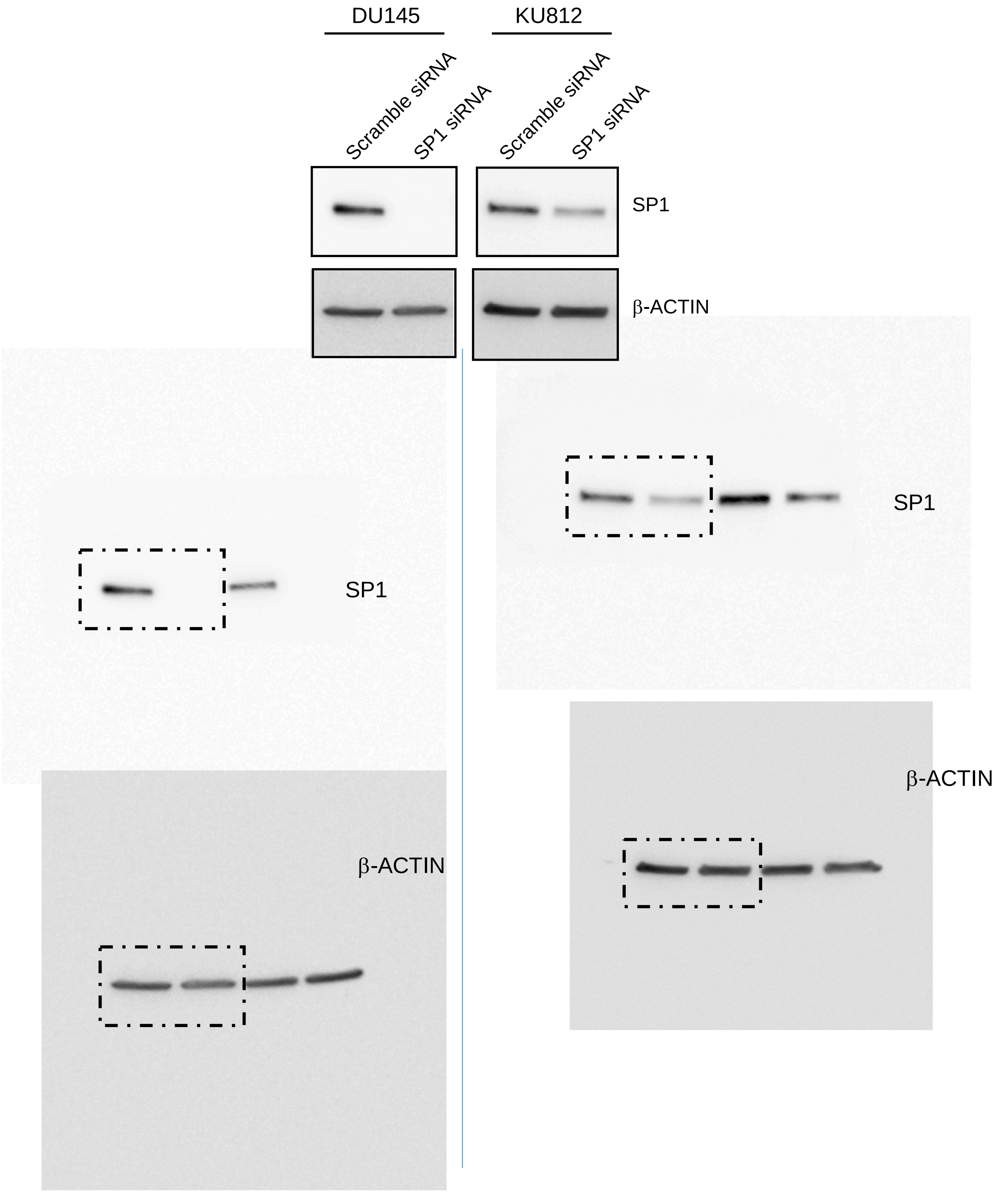

Supplementary Figure S3. **Full immunoblots for Figure 3f.** KU812 and DU145 cells were transfected with either scramble siRNA or SP1 siRNA (s13319, Ambion/ThermoFisher). 24 h after transfection (DU145 cells) or 48 h after transfection (KU812 cells), cells were lysed and 10 μg of total protein were used to detect SP1 levels by western-blotting followed by immuno-blotting with an anti-SP1 antibody (D4C3, Cell Signalling). Levels of β-Actin were used as a loading control. Dotted lines provide an indication of the cropped area.

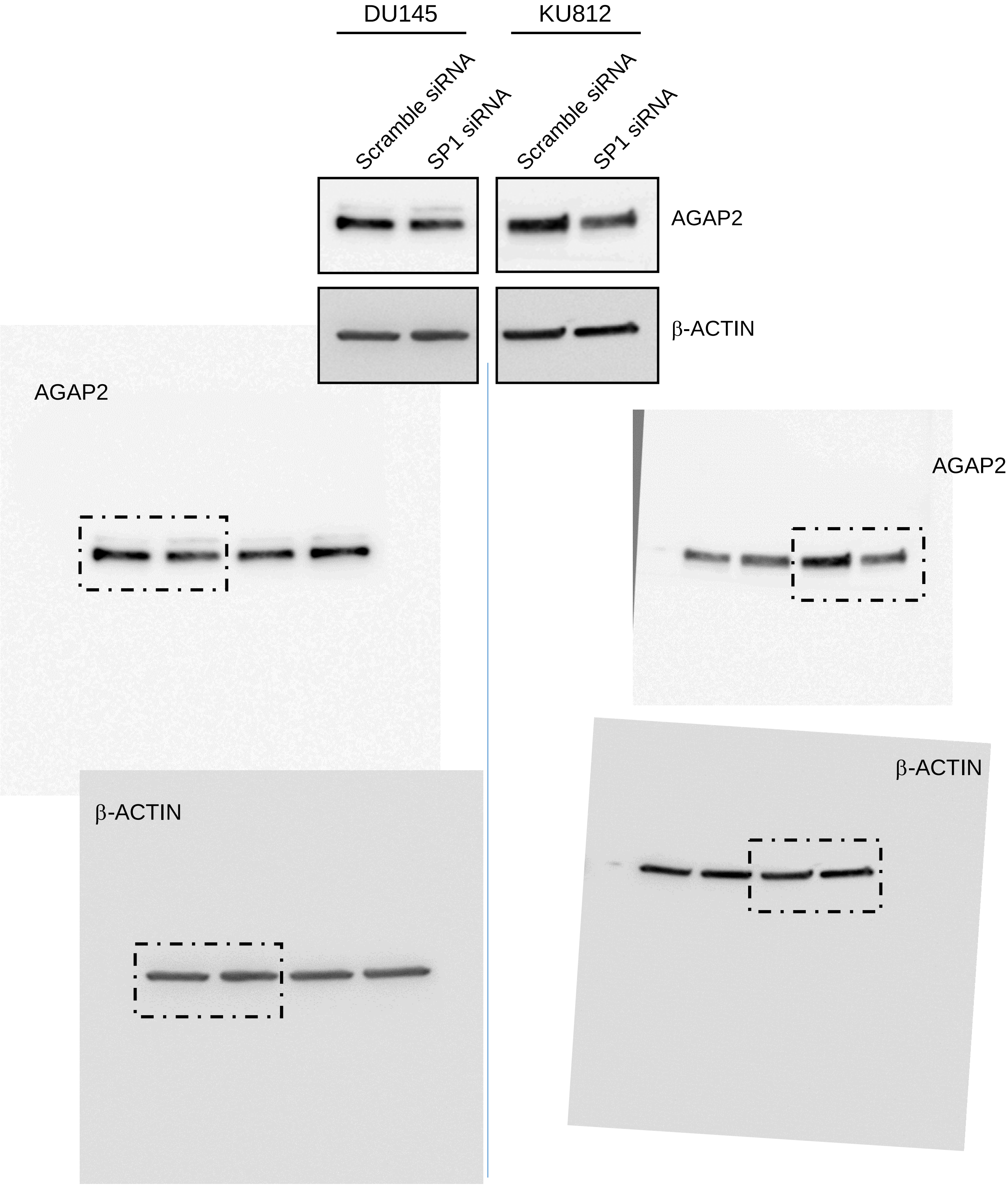

Supplementary Figure S4. **Full immunoblots for Figure 3g**. KU812 and DU145 cells were transfected with either scramble siRNA or SP1 siRNA (s13319, Ambion/ThermoFisher) and 24 h after transfection (DU145 cells) or 48 h after transfection (KU812 cells), cells were lysed and AGAP2 levels were detected by western-blotting followed by immuno-blotting with an anti-SP1 antibody (Cell Signalling). Levels of β-Actin were used as a loading control. Dotted lines provide an indication of the cropped area.

.

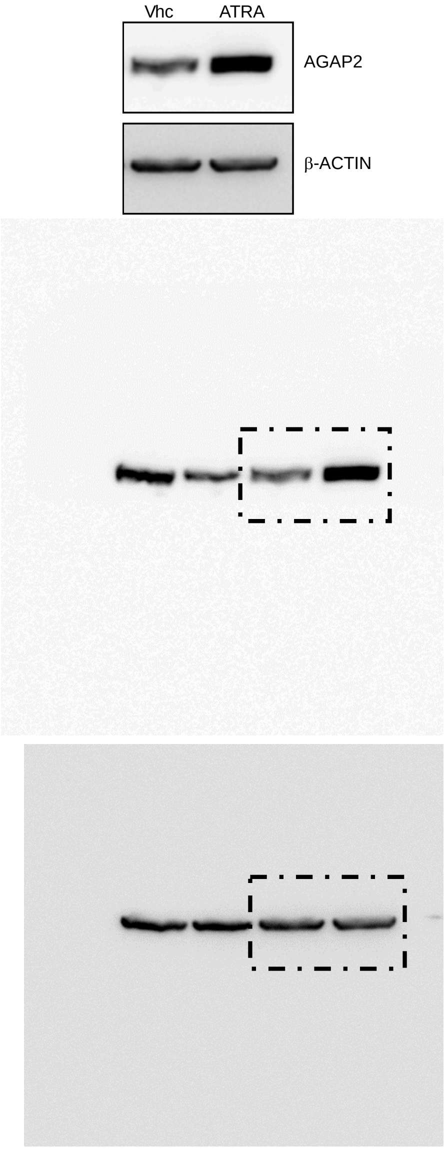

Supplementary Figure S5. **Full immunoblots for Figure 4e**. KU812 cells were grown overnight in clear RPMI medium with 5% charcoal-stripped serum. They were then treated with 1 μM ATRA for 24 h and lysed in RIPA buffer. 20 μg of protein were loaded on an SDS-PAGE, transferred to a nitrocellulose membrane and AGAP2 and β-Actin proteins were detected with specific antibodies. Dotted lines provide an indication of the cropped area.

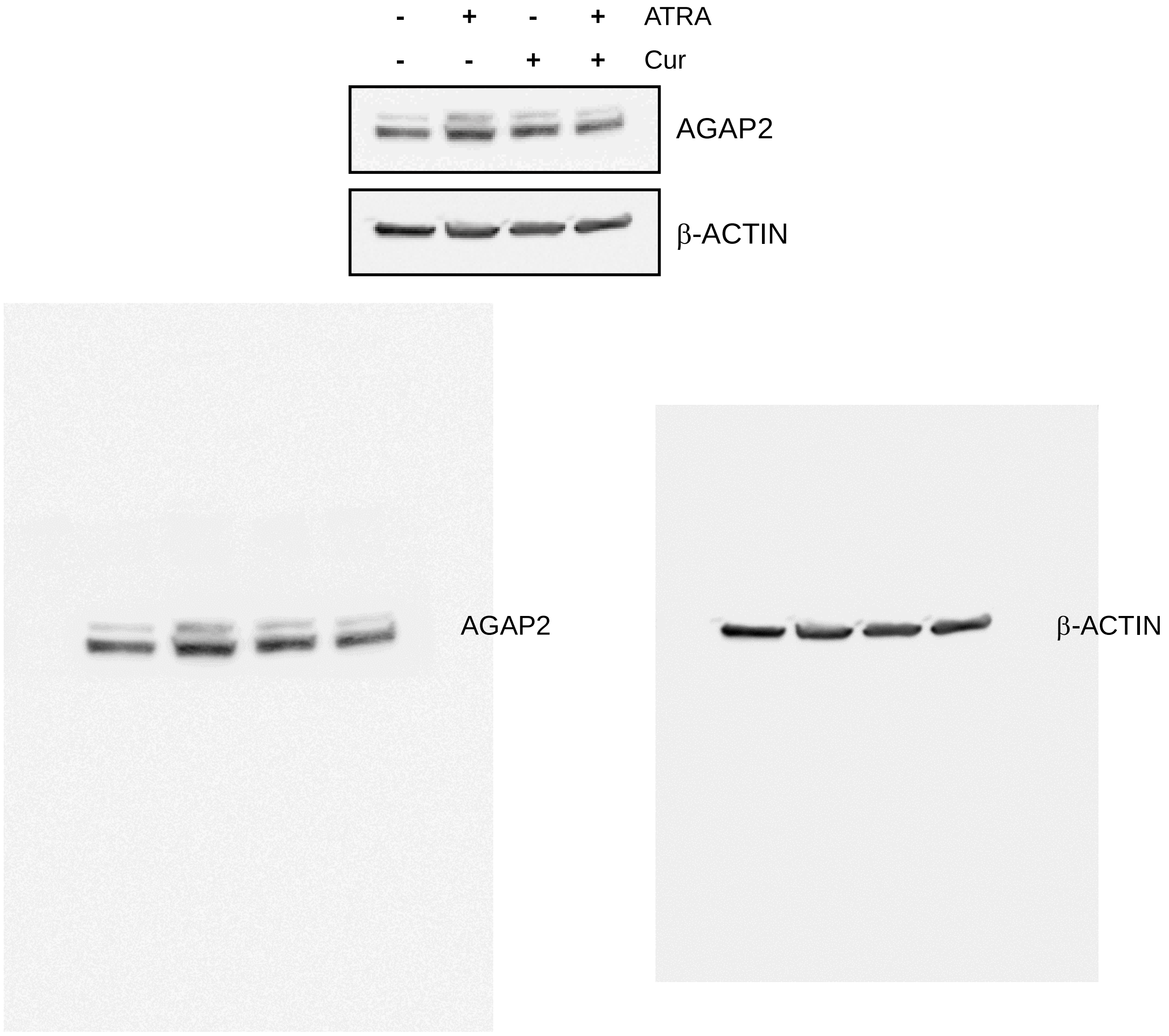

Supplementary Figure S6. **Full immunoblots for Figure 5c**. DU145 cells were grown overnight in clear DMEM medium with 5% charcoal-stripped serum. They were then treated with either 1 μM ATRA, 10 μM curcumin (Cur) or pre-treated with 10 μM curcumin for 1 h and then treated with 1 μM ATRA for 24 h and lysed in RIPA buffer. 20 μg of protein were loaded on an SDS-PAGE, transferred to a nitrocellulose membrane and AGAP2 and β-Actin protein levels were detected with specific antibodies.

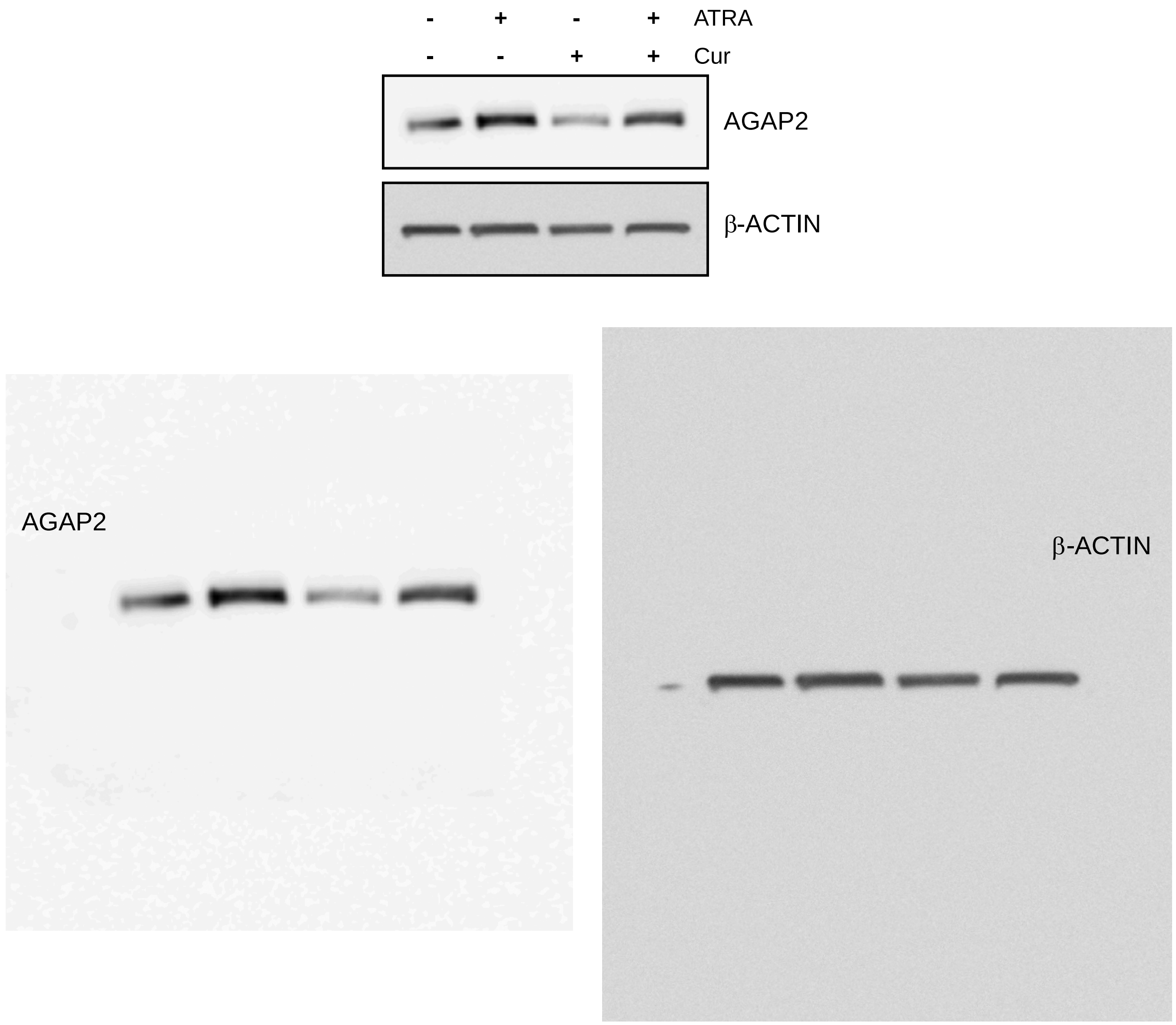

Supplementary Figure S7. **Full immunoblots for Figure 5d**. KU812 cells were grown overnight in clear RPMI medium with 5% charcoal-stripped serum. They were then treated with either 1 μM ATRA, 10 μM curcumin (Cur) or pre-treated with 10 μM curcumin for 1 h and then treated with 1 μM ATRA for 24 h and lysed in RIPA buffer. 20 μg of protein were loaded on an SDS-PAGE, transferred to a nitrocellulose membrane and AGAP2 and β-Actin protein levels were detected with specific antibodies.

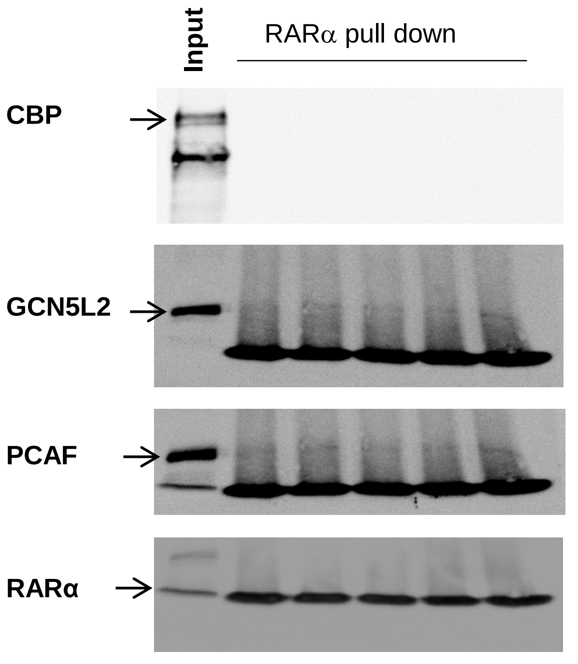

Supplementary Figure S8. **Lysine Acetyl transferases (KATs) associated to RARα in DU145 cells**. DU145 cells grown in the presence of serum were lysed in RIPA buffer and 500 μg of protein were use to immunoprecipitate RARα and its associated proteins using 2 μg of anti-RARα goat antibody (L-15, Sta. Cruz Biotechnology) and SureBeads Protein G (Bio-Rad). The immunocomplexes were separated in a SDS-PAGE and transferred to a nitrocellulose membrane . RARα protein expression (detected with rabbit antibody C-20, Sta. Cruz Biotechnology) is shown as a control. 50 μg of the total cell lysate was used as input control.
